# Efficacy and risk of cytotoxic chemotherapy in extensive disease-small cell lung cancer patients with interstitial pneumonia

**DOI:** 10.1186/s12885-019-5367-0

**Published:** 2019-02-20

**Authors:** Masayuki Shirasawa, Tomoya Fukui, Seiichiro Kusuhara, Yasuhiro Hiyoshi, Yoshiro Nakahara, Noriko Nishinarita, Satoshi Igawa, Katsuhiko Naoki

**Affiliations:** Department of Respiratory Medicine, Kitasato University School of Medicine, Kitasato University School of Medicine, 1-15-1 Kitasato, Minami-ku, Sagamihara, Kanagawa 252-0374 Japan

**Keywords:** Small cell lung cancer, Extensive disease, Cytotoxic chemotherapy, Interstitial pneumonia, Acute exacerbation

## Abstract

**Background:**

Small cell lung cancer (SCLC) is characterized by a high propensity for metastases and a poor prognosis irrespective of high sensitivity for initial chemotherapy. Although interstitial pneumonia (IP) is one of risk factors for lung cancer, efficacy of cytotoxic chemotherapy for patients with SCLC with IP remains unclear. Our study aims to evaluate the efficacy of systemic chemotherapy and assess risk of acute exacerbation (AE)-IP with cytotoxic drugs for extensive disease (ED)-SCLC patients with IP.

**Methods:**

We performed a retrospective study of 192 consecutive ED-SCLC patients with IP (*n* = 40) and without IP (*n* = 152) between 2008 and 2016.

**Result:**

31 of 40 ED-SCLC patients with IP and 130 of 152 patients without IP received systemic chemotherapy. The efficacy of chemotherapy in patients with IP was not inferior to that in patients without IP (overall survival [OS], 7.1 [95% confidence interval (CI): 0.2–14.0] vs. 10.0 [95% CI: 8.2–11.8] months, *P* = 0.57). Pretreatment serum levels of lactate dehydrogenase (LDH; 651.7 ± 481.0 vs. 301.4 ± 110.7 U/mL, *P* = 0.01) and C-reactive protein (CRP; 8.9 ± 9.6 vs. 1.8 ± 1.8 U/mL, *P* = 0.008) were correlated with developed AE-IP in the ED-SCLC patients with IP.

**Conclusion:**

Systemic chemotherapy was effective even in ED-SCLC patients with IP. However, the risk of developed AE-IP that was high in patients with IP and should be evaluated using serum LDH and CRP levels before initial chemotherapy.

**Electronic supplementary material:**

The online version of this article (10.1186/s12885-019-5367-0) contains supplementary material, which is available to authorized users.

## Background

Small cell lung cancer (SCLC) is characterized by a high propensity for metastases and a poor prognosis, despite it being one of the most chemosensitive solid tumors unlike other types of lung cancer [1]. 60–70% of patients with SCLC present with metastasis beyond a safe radiotherapy field that is defined as extensive disease (ED), and standard treatment for ED-SCLC patients is systemic chemotherapy with a median survival of about 8–12 months [2–5]. Generally, SCLC patients with interstitial pneumonia (IP) has been excluded in previous clinical studies, and efficacy of cytotoxic chemotherapy for ED-SCLC patients with IP is unclear.

Cigarette smoking and occupational exposures are common contributors not only to lung cancer, especially SCLC, but also to IP [6–9]. The prognosis of patients with IP is poor, and the mean duration from the diagnosis to death is nearly 3–5 years [10, 11]. In patients with IP, the incidence rate of lung cancer was reported to be approximately 17–48% [7–9]. On the other hand, approximately 15% of patients with lung cancer were diagnosed with IP [6]. Moreover, in patients with lung cancer with IP, acute exacerbation (AE)-IP was frequently reported after cytotoxic chemotherapy [12, 13]. and lung cancer with IP was considered as one of poor prognostic factors [14]. In clinical practice, lung cancer patients with IP are often recommended best supportive care (BSC) alone without systematic chemotherapy, because of high frequency of AE-IP [15].

Recently, it has been reported that the combination of platinum agents and etoposide for patients with ED-SCLC is feasible as first-line chemotherapy for patients with IP [16]. However, the safety and efficacy of cytotoxic chemotherapy for ED-SCLC patients with IP remain unclear in clinical settings. This study aims to assess the efficacy of cytotoxic chemotherapy and evaluate clinical factors associated with development of AE-IP with cytotoxic chemotherapy in ED-SCLC patients with IP.

## Methods

### Study patients and clinical data collection

This retrospective study enrolled 192 consecutive patients who were diagnosed with ED-SCLC between January 2008 and December 2016 at Kitasato University Hospital (Kanagawa, Japan), excluding 24 patients who participated in clinical trials and 3 patients who could not undergo pretreatment CT to diagnosis IP. Among the 192 patients, 161 received systemic chemotherapy, and 31 patients received BSC alone. Pre-existing IP was diagnosed when the diffuse ground-glass opacity, peripheral reticular opacity, consolidation without segmental distribution, and a honeycomb pattern were detected in bilateral lung fields on pretreatment CT findings. Acute exacerbation of IP is clinically defined according to published criteria as follows: (a) subjective progressive dyspnea within the last month; (b) new ground-glass opacities or consolidation observed on chest radiography and/or CT; (c) hypoxemia with a decline of 10 mmHg in the arterial oxygen pressure (PaO_2_) or more; and (d) the absence of infection, pulmonary embolism, congestive heart failure, or pneumothorax as a cause of acute worsening. In this study, we used the term “AE-IP” when patients with lung cancer developed acute respiratory diseases that satisfied the aforementioned definition of acute exacerbation of IP after chemotherapy [17–20]. In ED-SCLC patients without IP, several patterns of drug-induced interstitial lung diseases exist, ranging from benign infiltrates to life-threatening acute respiratory distress syndrome. However, ED-SCLC patients with IP may develop the acute exacerbation of pre-existing IP induced by cytotoxic drugs. In this study, since it is difficult to distinguish between the causes of AE-IP, we used the term “AE-IP” when ED-SCLC patients developed acute respiratory conditions that satisfied the abovementioned definition of AE-IP after chemotherapy.

### Evaluation of response and toxicity

We classified the tumor response in accordance with the Response Evaluation Criteria for Solid Tumors (version 1.1) based on the results of a complete medical history, physical examination, chest X-ray, CT of chest and abdomen, and other procedures, such as head MRI, PET, and bone scintigraphy. In addition, adverse events were recorded and graded according to the Common Terminology Criteria for Adverse Events version 4.0 (CTCAE v4.0).

### Statistical analyses

Differences of clinical and laboratory data between two groups were tested using Mann–Whitney *U*-test. We analyzed categorical data with *χ*^2^ test. Survival was evaluated from start of chemotherapy to date of documentation of treatment failure (death or disease progression) or date of censoring at final follow-up examination. All survival analyses were performed using Kaplan–Meier method. In addition, survival between subgroups based on predictive factors was compared using a log-rank test. We used a Cox proportional hazards model for univariate and multivariate analyses to identify the prognostic factors. All analyses were performed using the SPSS software program, version 23.0 (SPSS Inc., Chicago, IL).

This study was approved by the Kitasato University Medical Ethics Organization (B17–253).

## Results

### Patient characteristics

Main clinical characteristics of patients are shown in Table 1. Among 192 patients, 163 (85%) were males, and the median age was 72 (range: 42–95) years. Among all patients with ED-SCLC, 40 (21%) were diagnosed with IP. We observed no difference in the sex, smoking status, serum levels of albumin, lactate dehydrogenase (LDH), and C-reactive protein (CRP) between the SCLC patients with IP and those without IP. The serum KL-6 levels (832.0 ± 701.2 vs. 437.2 ± 480.3 U/mL; *P* < 0.001) and SP-D levels (141.0 ± 91.0 vs. 62.6 ± 47.6 U/mL; *P* = 0.005) were higher in ED-SCLC patients with IP than in those without IP.

**Table 1:** Patient characteristics in this study (*n* = 192)

	Without IP	With IP	P*
	*n* = 152	*n* = 40	
Age, median (range), years, *n* (%)	71 (42–95)	73 (47–83)	0.82
< 75 years	98 (64.5)	25 (62.5)	
≥75 years	54 (35.6)	15 (37.5)	
Gender, *n* (%)			
Male	126 (82.9)	37 (92.5)	0.13
Female	26 (17.1)	3 (7.5)	
Smoking status, *n* (%)			
Never	8 (5.5)	2 (5.0)	0.94
Former/Current	139 (91.4)	37 (92.5)	
ECOG PS, *n* (%)			
0/1	80 (52.6)	20 (50.0)	0.77
2/3/4	72 (47.4)	21 (50.0)	
Blood tests, mean ± SD			
Alb, g/dL	3.6 ± 0.5	3.6 ± 0.4	0.35
LDH, IU/L	476.7 ± 778.4	391.5 ± 293.5	0.72
CRP, mg/dL	3.0 ± 5.0	3.3 ± 5.5	0.45
KL-6, U/mL	437.2 ± 480.3	832.0 ± 701.2	< 0.001
SP-D, U/mL	62.6 ± 47.6	141.0 ± 91.0	0.005

* *p*-values were analyzed by the χ^2^ test

Note: *IP* interstitial pneumonia, *PS* performance status, *SD* standard deviation, *Alb* albumin, *LDH* lactate dehydrogenase, *CRP* C-reactive protein, *KL-6* Krebs von den Lungen-6, *SP-D* pulmonary surfactant protein-D

### Treatment outcomes

**Fig. 1:**
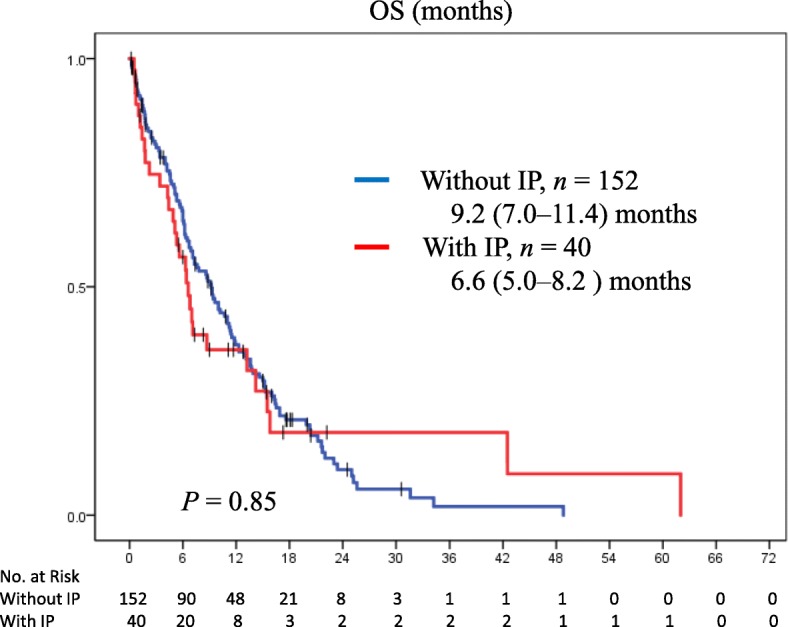
The Kaplan–Meier analysis of OS in patients without IP (blue) vs. patients with IP (red). *P* values were determined by the log-rank test; the number of individuals in each group and median survival time (95% CI) are indicated

Median overall survival **(**OS) of all patients (*n* = 192) was 8.6 (95% confidence interval [CI]: 6.8–10.4) months, and the OS in patients with SCLC with IP was not inferior to that in patients without IP (6.6 [95% CI: 5.0–8.2] vs. 9.2 [95% CI: 7.0–11.4] months; *P* = 0.85; Fig. 1). The percentage of patients with and without IP who received BSC alone was 23% (*n* = 9 of 40) and 15% (*n* = 22 of 152), respectively. Eleven of 31 patients with IP (36%) and 73 of 129 patients without IP (57%) received subsequent chemotherapy (*P* = 0.20).

**Fig. 2:**
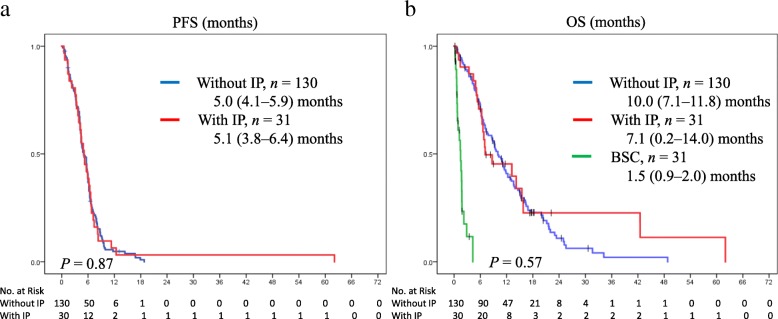
The Kaplan–Meier analyses of the PFS (**a**) and OS (**b**) for patients without IP (blue) vs. patients with IP (red) who were treated with chemotherapy. The OS of patients who only received BSC (**b**). The median OS of all patients was 9.6 (95% CI: 7.8–11.4) months. *P* values were determined by the log-rank test; the number of individuals in each group and median survival time (95% CI) are indicated

Among the patients who received chemotherapy (*n* = 161), no difference was observed in response rates between the IP group and non-IP group (62% vs. 65%; *P* = 0.76; Table 2). Median progression-free survival (PFS) on the first-line chemotherapy demonstrated no significant differences between patients with and without IP (5.0 [95% CI: 4.1–5.9] vs. 5.1 [95% CI: 3.8–6.4] months; *P* = 0.87; Fig. 2a). Median OS of all patients was 9.6 (95% CI: 7.8–11.4) months, and the OS in patients with SCLC with IP was also not inferior to that in patients without IP (7.1 [95% CI: 0.2–14.0] vs. 10.0 [95% CI: 8.2–11.8] months; *P* = 0.57; Fig. 2b).

**Table 2:** The efficacy of chemotherapy in patients with ED-SCLC without or with IP (*n* = 161)

	Without IP	With IP,
	*n* = 130	*n* = 31
Response to initial chemotherapy, *n* (%)		
Partial response	80 (61.5)	20 (64.5)
Stable disease	20 (15.4)	6 (19.4)
Progressive Disease	17 (13.1)	2 (6.4)
Not evaluated	13 (10.0)	3 (9.7)
Number of chemotherapy, *n* (%)		
1	53 (40.8)	20 (64.5)
2	46 (35.4)	10 (32.3)
≥3	31 (23.8)	1 (3.2)
Regimen of initial chemotherapy, *n* (%)		
CDDP + CPT	17 (13.1)	0 (0)
CDDP + ETP	5 (3.8)	6 (19.4)
CBDCA + CPT	4 (3.1)	0 (0)
CBDCA + ETP	35 (26.9)	21 (67.7)
AMR	69 (53.1)	4 (12.9)

Note: *IP* interstitial pneumonia, *CDDP* cisplatin, *CBDCA* carboplatin, *CPT* irinotecan, *ETP* etoposide, *AMR* amrubicin

In the univariate survival analysis of patients with ED-SCLC who received chemotherapy, poor PS (hazard ratio [HR], 1.61; 95% CI: 1.13–2.29; *P* = 0.01) and the use of non-platinum regimen as the first-line chemotherapy (HR, 0.60; 95% CI: 0.42–0.86; *P* = 0.005) were unfavorable prognostic factors (Table 3). Based on the results of multivariate analysis, poor PS (HR, 1.45; 95% CI: 0.99–2.12; *P* = 0.06) was confirmed as an independent unfavorable prognostic factor, but pre-existing IP was not associated (HR, 1.00; 95% CI: 0.59–1.70; *P* = 0.99) (Table 3).

**Table 3:** Univariate and multivariate analyses for overall survival in ED-SCLC patients who received chemotherapy (*n* = 161)

	Univariate analysis			Multivariate analysis		
Factors	HR	95% CI	*P*	HR	95% CI	*P*
IP, yes vs. no	0.87	0.54–1.40	0.56	1.00	0.59–1.70	0.99
Sex, female vs. male	1.08	0.69–1.69	0.73	Excluded		
Age, ≧75 vs. < 75 years	1.24	0.85–1.80	0.26	1.15	0.77–1.71	0.49
ECOG PS 2–4 vs. 0–1	1.61	1.13–2.29	0.01	1.45	0.99–2.12	0.06
Smoking, yes vs. no	1.15	0.50–2.61	0.75	Excluded		
Platinum doublet vs AMR	0.60	0.42–0.86	0.005	0.70	0.46–1.06	0.09

Note: *IP* interstitial pneumonia, *PS* performance status, *AMR* amrubicin, *HR* hazard ratio, *95% CI* 95% confidence interval

### Clinical factors associated with AE-IP

In 31 patients who had ED-SCLC with IP and administrated cytotoxic drugs, 7 (23%) patients developed AE-IP in course of chemotherapy, with a higher incidence of AE-IP compared to those without IP (*n* = 1 of 130; < 1%; *P* <  0.001). Among 7 patients with AE-IP, 4 patients received combination chemotherapy with platinum and etoposide, and 3 received amrubicin monotherapy (Table 4). In patients with developed AE-IP, the frequency of poor PS (*P* = 0.004) and amrubicin treatment (*P* = 0.005) was significantly higher compared with patients who did not developed AE-IP. The pretreatment serum LDH (651.7 ± 482.0 vs. 301.4 ± 110.7 U/mL; *P* = 0.01) and CRP (8.9 ± 9.6 vs. 1.8 ± 1.8 U/mL; *P* = 0.008) were higher in patients with IP who developed AE-IP than in those who did not develop AE-IP with IP (Table 4). Pretreatment serum KL-6 (1104.0 ± 555.1 vs. 823.8 ± 821.0 U/mL; *P* = 0.09) levels and SP-D (221.7 ± 118.7 vs. 120.5 ± 78.1; *P* = 0.11) levels of patients with AE-IP tended to be higher than that of patients without AE-IP. There was no statistically significant difference in values between those who did and did not experience AE-IP.

**Table 4:** Characteristics of patients with ED-SCLC and IP (*n* = 31)

	D-ILD (+)	D-ILD (−)	P*
	*n* = 7	*n* = 24	
Age, median (range), years, *n* (%)	70 (62–76)	74 (47–82)	
< 75 years	1 (14.3)	15 (62.5)	0.25
≥75 years	6 (85.7)	9 (37.5)	
Gender, *n* (%)			
Male	6 (85.7)	22 (91.7)	0.64
Female	1 (14.3)	2 (8.3)	
Smoking status, *n* (%)			
Never	1 (14.3)	1 (4.2)	0.36
Former/Current	6 (85.7)	22 (91.7)	
ECOG PS, *n* (%)			
0/1	1 (14.3)	18 (75)	0.004
2/3/4	6 (85.7)	6 (25)	
Blood testss, mean ± SD			
Alb, g/dL	3.2 ± 0.5	3.7 ± 0.4	0.03
LDH, IU/L	651.7 ± 482.0	301.4 ± 110.7	0.01
CRP, mg/dL	8.9 ± 9.6	1.8 ± 1.8	0.008
KL-6, U/mL	1104.0 ± 555.1	823.8 ± 821.0	0.09
SP-D, U/mL	221.7 ± 118.7	120.5 ± 78.1	0.11
Regimen of initial chemotherapy, *n* (%)			
CDDP + ETP	0 (0)	5 (20.8)	0.005*
CBDCA + ETP	4 (57.1)	18 (75)	
AMR	3 (42.9)	1 (4.2)	

* *p*-values were analyzed by the χ^2^ test

Note**:** *D-ILD* drug-induced interstitial lung disease, *PS* performance status, *SD* standard deviation, *Alb* albumin, *LDH* lactate dehydrogenase, *CRP* C-reactive protein, *KL-6* Krebs von den Lungen-6, *SP-D* pulmonary surfactant protein-D, *CDDP* cisplatin, *CBDCA* carboplatin, *ETP* etoposide, *AMR* amrubicin

## Discussion

This study suggested that systemic chemotherapy was a treatment option in patients with ED-SCLC with IP, although those patients had a high risk of developing AE-IP by cytotoxic drugs. To the best of our knowledge, this is first report demonstrating that high serum concentrations of LDH and CRP before initiating chemotherapy are unfavorable predictive factors for developing AE-IP in patients with ED-SCLC with IP. Togashi et al., reported that patients with SCLC complicated with IP were related with a prognostic factor [14]. In their cohort, 53 of 122 patients were limited disease SCLC, for whom standard treatment is chemoradiotherapy. Chemoradiotherapy was not recommended for patients with IP to prevent acute exacerbation of IP. It is possible that differences in treatment affected prognosis. In our study, we analyzed 192 ED-SCLC and evaluated the efficacy of chemotherapy. We observed similar survival benefit between ED-SCLC with IP and that without IP. We suggest that systematic chemotherapy is a treatment option even for patients with ED-SCLC combined with IP.

When administering chemotherapy for SCLC patients with IP, clinicians must be wary of developing AE-IP, because patients with AE-IP had demonstrated high mortality in previous studies [13, 17, 21, 22]. In our study, 7 of 31 patients (23%) with ED-SCLC with IP developed AE-IP. Regarding association between chemotherapy regimen and AE-IP, previous studies have revealed that amrubicin and irinotecan were associated with a high incidence of AE-IP in patients with IP [23, 24]. In addition, a combination chemotherapy with platinum and etoposide is reported as the preferable first-line chemotherapy regimen for patients with SCLC with IP [16, 17]. In our study, a majority of patients with IP (*n* = 28 of 31) received carboplatin plus etoposide in the all courses of chemotherapy, and 4 (14%) patients developed AE-IP, similar to those previously reported [13, 17]. Although amrubicin is a potential agent for the treatment of ED-SCLC as a second-line setting, [25, 26] high incidence of AE-IP induced by amrubicin was reported among SCLC patients with IP, [23] so AE-IP associated with amrubicin is an issue. In all courses of chemotherapy in our study, 8 patients with IP received amrubicin, and 3 patients (38%) developed AE-IP. On the other hand, 111 patients without IP received amrubicin, and 1 patient (1%) developed AE-IP. This data suggested a high incidence of AE-IP in patients with ED-SCLC with IP treated with amrubicin. For ED-SCLC patients with IP, a combination chemotherapy with platinum and etoposide in the first-line chemotherapy is preferable to prevent developing AE-IP.

The pathogenesis of AE-IP by cytotoxic drugs is poorly understood. However, most toxic effects are thought to result from direct cytotoxicity. Active oxygen or either a growth factor, inflammatory cytokine or vascularization factor localized in a part of the lung plays an important role in inducing inflammation, and it is also known that values for these factors increase temporarily after chemotherapy [27]. Inflammation induced by these factors is considered one of the reasons underlying the development of AE-IP. The increase in the levels of these cytokines may correlate with IP activity and, thus, with active inflammation [28, 29]. KL-6 and SP-D are known sensitive biomarkers for IP, but there is no consensus of predictive markers for AE-IP although these have been reported previously [13, 30]. We investigated the significance of KL-6 and SP-D as predictors of AE-IP in ED-SCLC patients treated with chemotherapy and found no statistically significant difference between those who did and did not experience acute exacerbation. Minegishi et al. reported that a high serum concentration of CRP before initiation of chemotherapy was associated with a significant likelihood of a patient developing AE-IP[13]. By comparing between patients with IP who had AE-IP and patients with IP who did not have AE-IP, we demonstrated that the pretreatment serum levels of LDH and CRP were significantly higher in ED-SCLC patients with IP who developed AE-IP than those who did not develop AE-IP. Among seven patients developed AE-IP, six patients had high LDH (> 275 IU/L; median in ED-SCLC patients with IP) and six patients had high CRP (> 1.51 mg/dl; median in ED-SCLC patients with IP). It was indicated that a high serum concentration of LDH or CRP before initiation of chemotherapy was associated with a significant likelihood of a patient with IP developing AE-IP by cytotoxic drugs (LDH: sensitivity 87%, specificity 63%; CRP: sensitivity 87%, specificity 58%). It is possible that high inflammatory IP is highly activated due to stimulation of cytotoxic drugs and is more likely to cause AE-IP, but the details remain unclear.

In our study, approximately 69 (53.1%) patients without IP and 4 (12.9%) patients with IP received AMR in the initial chemotherapy. Among ED-SCLC without IP, AMR was selected for elderly patients and those with renal dysfunction, SVC syndrome, and poor PS in our clinical practice; thus, ED-SCLC patients without IP might have relatively poor OS compared to patients in previous clinical trials [30]. Additionally, we compared the OS of ED-SCLC patients with and without IP between the group that received platinum doublet or the group that underwent AMR (Additional file 1: Figure S1). Among ED-SCLC patients who received platinum-based chemotherapy in the initial chemotherapy, the OS of patients with IP (*n* = 27) was similar to the OS of those without IP (*n* = 60) (11.8 [95% CI: 8.9–14.7] vs. 8.7 [95% CI: 0.4–17.0] months; *P* = 0.93; Additional file 1: Figure S1-a). Regardless of the type of initial chemotherapy, the prognosis of ED-SCLC patients with IP may be somewhat poor compared with the prognosis of those without IP. Less choices of therapeutic agents and occurrence of AE-IP can be attributed to this difference. In our study, secondary chemotherapy transfer rates were 59.2 and 35.5% for patients without and with IP, respectively. Among ED-SCLC patients with IP, patients who developed AE-IP showed a poor significantly prognosis compared with those who did not develop AE-IP (4.4 [95% CI: 1.0–7.8] vs. 13.2 [95% CI: 2.1–24.3] months; *P* = 0.020; Additional file 2: Figure S2). The OS of ED-SCLC patients with IP who did not develop AE-IP was not different from the prognosis of ED-SCLC patients without IP (10.0 [95% CI: 8.2–11.8] months; *P* = 0.173; Additional file 2: Figure S2). Therefore, preventing AE-IP is crucial, and the activity of IP combined with ED-SCLC should be validated using pretreatment markers, such as levels of LDH and CRP, for assessing the risk of developing AE-IP. Early recognition and symptomatic support of acute exacerbation may improve outcomes in these patients.

Finally, this study has certain limitations. First, this was a retrospective study conducted in a single institution; hence, the results cannot be completely regarded as definitive. Second, all our patients were diagnosed with IP using HRCT imaging and laboratory findings, but not based on pathological findings. In the clinical practice, diagnosis of the AE-IP based on clinical and radiological findings is challenging. Thus, we cannot entirely exclude the possibility that our patients had developed lymphangitic carcinomatosis, pneumonitis, various infectious diseases, congestive heart failure, thromboembolism or some other disease, rather than AE-IP.

## Conclusion

Our retrospective study supported the survival benefit of cytotoxic chemotherapy for ED-SCLC patients with IP. However, cytotoxic chemotherapy caused developing AE-IP in some patients with IP. Therefore, we should validate the activity of IP combined with lung cancer for a developing risk of AE-IP using pretreatment levels of LDH and CRP.

## Additional files

Additional file 1: **Figure S1.** Kaplan–Meier analyses of overall survival (OS) of patients without IP (blue) vs. patients with IP (red) who were treated with platinum doublet (1-a) and amrubicin (1-b). *P* values were determined by log-rank test; the number of individuals in each group and median survival time (95% CI) are indicated. (PPTX 61 kb)

Additional file 2: **Figure S2.** Kaplan–Meier analyses of overall survival (OS) of ED-SCLC patients treated with chemotherapy. Blue: Patients without IP who did not develop AE-IP; Red: Patients with IP who did not develop AE-IP; Orange: Patients who developed AE-IP; and Green: the OS of ED-SCLC patients who received the best supportive care (BSC) only. The number of individuals in each group and median survival time (95% CI) are indicated. (PPTX 52 kb)

## Abbreviations

AE-IP: Acute exacerbation- interstitial pneumonia
BSC: Best supportive care
CI: Confidential interval
CRP: C-reactive protein
ED: Extensive-disease
IP: Interstitial pneumonia
KL-6: Krebs von den Lungen-6
LDH: Lactate dehydrogenase
OS: Overall survival
PFS: Progression free survival
SCLC: Small cell lung cancer
SP-D: pulmonary surfactant protein-D
