# Supplementary material for: Efficacy and risk of cytotoxic chemotherapy in extensive disease-small cell lung cancer patients with interstitial pneumonia
Source: BMC Cancer. 2019 Feb 20;19:163. doi: 10.1186/s12885-019-5367-0 (PMC6391765; doi:10.1186/s12885-019-5367-0)
Supplement: Supplementary file 1 — Figure S1. Kaplan–Meier analyses of overall survival (OS) of patients without IP (blue) vs. patients with IP (red) who were treated with platinum doublet (1-a) and amrubicin (1-b). P values were determined by log-rank test; the number of individuals in each group and median survival time (95% CI) are indicated. (PPTX 61 kb) [file 12885_2019_5367_MOESM1_ESM.pptx]

## Slide 1
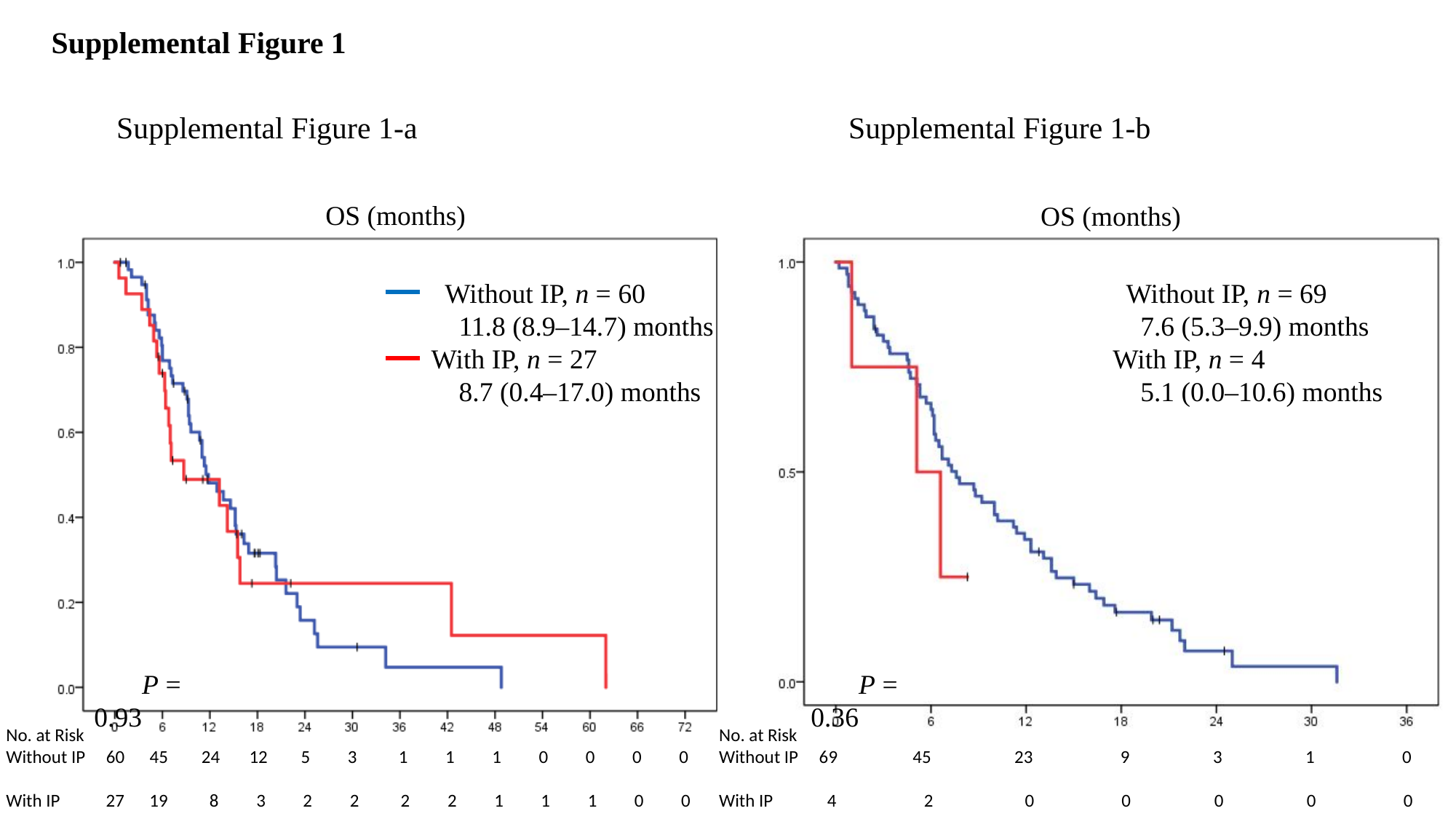

Supplemental Figure 1
Supplemental Figure 1-a
Supplemental Figure 1-b
OS (months)
OS (months)
　 Without IP, n = 60
 11.8 (8.9–14.7) months
 With IP, n = 27
 8.7 (0.4–17.0) months
　 Without IP, n = 69
 7.6 (5.3–9.9) months
 With IP, n = 4
 5.1 (0.0–10.6) months
　 P = 0.93
　 P = 0.36
No. at Risk
Without IP 60 45 24 12 5 3 1 1 1 0 0 0 0
With IP 27 19 8 3 2 2 2 2 1 1 1 0 0
No. at Risk
Without IP 69 45 23 9 3 1 0
With IP 4 2 0 0 0 0 0
