# Supplementary material for: Efficacy and risk of cytotoxic chemotherapy in extensive disease-small cell lung cancer patients with interstitial pneumonia
Source: BMC Cancer. 2019 Feb 20;19:163. doi: 10.1186/s12885-019-5367-0 (PMC6391765; doi:10.1186/s12885-019-5367-0)
Supplement: Supplementary file 2 — Figure S2. Kaplan–Meier analyses of overall survival (OS) of ED-SCLC patients treated with chemotherapy. Blue: Patients without IP who did not develop AE-IP; Red: Patients with IP who did not develop AE-IP; Orange: Patients who developed AE-IP; and Green: the OS of ED-SCLC patients who received the best supportive care (BSC) only. The number of individuals in each group and median survival time (95% CI) are indicated. (PPTX 52 kb) [file 12885_2019_5367_MOESM2_ESM.pptx]

## Slide 1
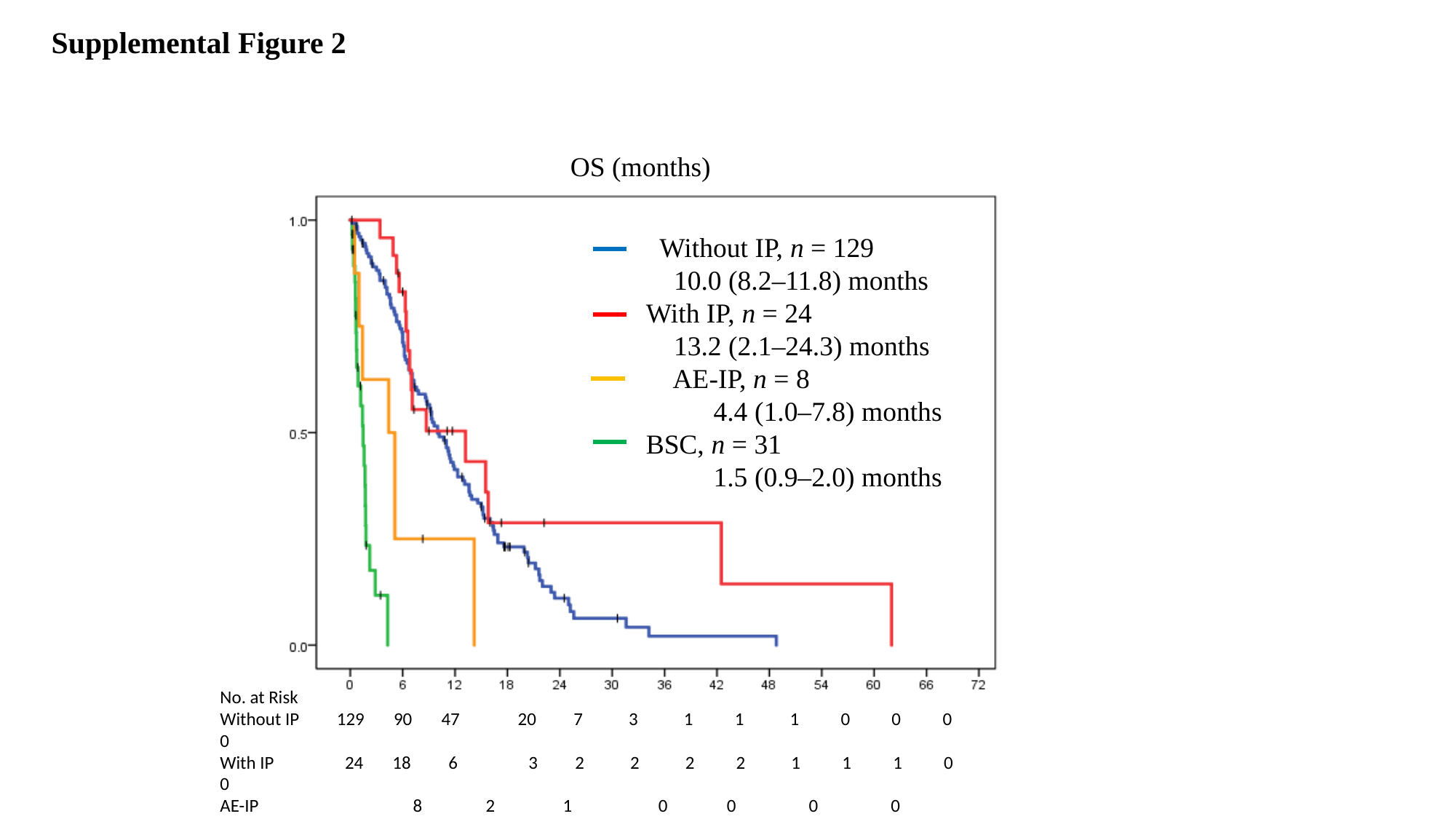

Supplemental Figure 2
OS (months)
　 Without IP, n = 129
 10.0 (8.2–11.8) months
 With IP, n = 24
 13.2 (2.1–24.3) months
　　AE-IP, n = 8
 　　　4.4 (1.0–7.8) months
 BSC, n = 31
 　　　1.5 (0.9–2.0) months
No. at Risk
Without IP 129 90 47 　 20 7 3 1 1 1 0 0 0 0
With IP 24 18 6 　 3 2 2 2 2 1 1 1 0 0
AE-IP　　　　　　　　8　　　2　　　 1　　　　 0　 　 0　　　 0　　　 0　　　 0　　　 0　　　 0 　　 0　　　 0　　　 0
BSC 　　　　 　31　　 0　 　 0　　 　 0　　 0　　　 0　　　 0　　　 0　　　 0　　　 0　　　 0　　　 0　　　 0
